# Blocking the survival of the nastiest by HSP90 inhibition

**DOI:** 10.18632/oncotarget.6971

**Published:** 2016-01-21

**Authors:** Paul Workman, Paul A. Clarke, Bissan Al-Lazikani

**Affiliations:** ^1^ Cancer Research UK Cancer Therapeutics Unit, Division of Cancer Therapeutics, The Institute of Cancer Research, London, UK

**Keywords:** HSP90

## Abstract

It is now recognised that genetic, epigenetic and phenotypic heterogeneity within individual human cancers is responsible for therapeutic resistance – knowledge that is having a profound impact on current thinking and experimentation. There has been concern that molecularly targeted therapy is doomed to failure, with resistant clones emerging in response to the Darwinian selective pressure of any drug treatment. However, two studies have shown that the evolution of drug resistance can be restrained by co-administration of a pharmacologic inhibitor of the HSP90 molecular chaperone.

It has been known for decades that individual cancers are heterogeneous, undergo progression to increasingly malignant and aggressive forms and commonly develop therapeutic resistance. What has changed is our ability to elucidate this progression in extraordinary molecular detail – and especially to characterise large numbers of human cancers using techniques such as next-generation sequencing. Another significant development has been the synthesis of a new and sophisticated conceptual framework for cancer evolution, which has enabled a more comprehensive and nuanced understanding of disease progression. As a result of this emerging framework, therapeutic manoeuvres have been suggested that can directly impact on patient treatment and inform our thinking about future therapeutic strategies [1, 2].

Importantly, we now recognize that drug resistance is an enduring feature of the cancer state that applies not only to first generation cytotoxic drugs but also to the new generation of sophisticated molecularly targeted agents that exploit oncogene addiction, synthetic lethality and so on [3].

In the 1860s, Herbert Spencer and Charles Darwin first articulated the term ‘survival of the fittest’ to describe the evolution of species by selection for heritable traits that enable adaptation to the local environment. Here, by analogy, we refer to this malign exemplar of the evolutionary paradigm in cancer as the ‘survival of the nastiest’ [http://www.theguardian.com/science/2013/aug/25/hiv-aids-cancer]. There are clear parallels between the evolution of resistance in individual cancers and the emergence of antibiotic-resistant micro-organisms, including the use of combinatorial drug therapy to counteract the problem [3].

The contemporary view of cancer evolution can be traced to 1902 and Theodor Boveri, who was the first to propose that the origins of malignancy lie in chromosomal abnormalities that are passed on to daughter cells. Boveri's theory of the clonal ancestry, and of the progression of cancer driven by acquired genetic instability, was subsequently supported by a large body of work – initially using cytogenetic and protein biomarkers – as articulated in a landmark article by Peter Nowell in 1976 [4].

In recent years, high-resolution molecular analysis of patients' tumors by single-cell sequencing, and other sophisticated techniques, has yielded astonishing insights into the degree of spatial and temporal variation in subclonal cancer populations. It has uncovered a branched pattern of cancer development where different mutations can arise in distinct subpopulations (branches) of the same tumor, generating resistance against individual molecularly targeted drugs [2] (Figure 1A). Conversely, through parallel evolution, distinct clones may converge on an identical driver gene or signaling pathway, thereby increasing the likelihood of a durable response to a single targeted agent [2]. Evidence also indicates that cancers may evolve gradually (microevolution), that is in a stepwise fashion through point mutations, or dramatically (macroevolution) via large chromosomal rearrangements or genome doublings [2].

**Figure 1:**
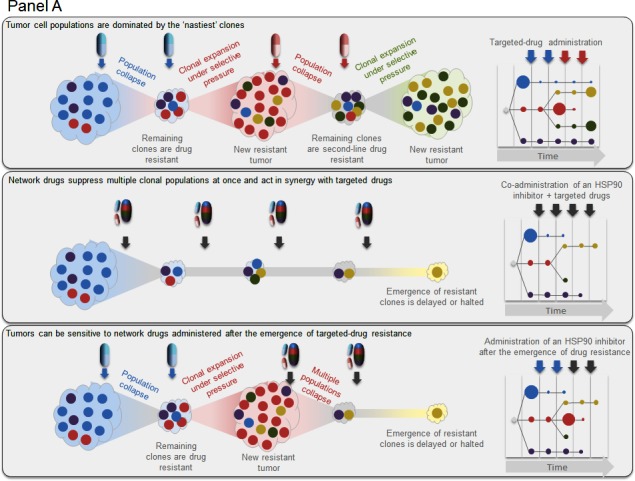

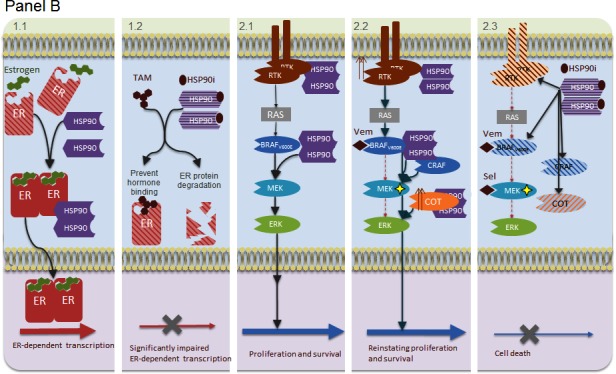
HSP90 inhibitors block the emergence of drug resistance in mouse models of human cancer Panel **A**: The emergence of more malignant and aggressive clones is driven by genetic instability and clonal evolution in response to the selective pressure of drug treatment. This leads to drug resistance [1, 2]. The administration of network drugs, such as HSP90 inhibitors, can delay or suppress the emergence of resistance to targeted drugs. Panel **B**: Mechanistic studies show that resistance to estrogen receptor (ER) antagonists and the BRAF inhibitor vemurafenib (Vem) can be blocked or delayed by co-administration of an HSP90 inhibitor (HSP90i) [6, 7]. Panel B1.1: HSP90 is important for estrogen-ER binding and thus ER activation, as well as ER stability. Panel B1.2: Combining an HSP90i with the ER antagonist 4-hydroxytamoxifen (TAM) prevents estrogen binding and promotes ER degradation, thus prolonging the anti-tumor effect. Panel B2.1: The BRAFV600E-mutant protein requires HSP90 for its stability and function. Panel B2.2: Cells rapidly acquire resistance to the BRAF inhibitor Vem by upregulating other components of the signaling pathway (e.g. RTK, COT kinase), through the heterodimerisation of BRAFV600E with CRAF, or by acquiring mutations in MEK. Panel B2.3: HSP90i treatment can overcome acquired resistance to Vem or the MEK inhibitor selumetinib (Sel) by disrupting multiple resistance mechanisms. Co-administration of an HSP90 inhibitor may be of benefit because of: 1) an additional effect on the target of the anti-estrogen or kinase inhibitor (ie. ER or BRAF); 2) an effect on alternative oncogenic targets or pathways which would otherwise lead to resistance; or 3) beneficial effects on the tumor microenvironment. Integrative molecular analysis, particularly detailed genomic sequencing and protein biomarker profiling before, during and after treatment, is needed to establish the molecular mechanisms involved. Future combination treatments may also feature immune therapy.

How then might inhibitors of the HSP90 molecular chaperone block cancer evolution and overcome drug resistance? Binding to HSP90 is essential for the activity and stability of many oncogenic proteins – especially those activated by mutation or translocation, or that are overexpressed. Small molecule HSP90 inhibitors exploit the fact that oncogenic driver proteins rely on molecular chaperones for stability and function, and several HSP90 inhibitors are now undergoing clinical trial [5]. Previously, it was hypothesized that HSP90 inhibitors could overcome resistance to established drugs by disrupting multiple oncoproteins, signaling pathways and hallmark traits simultaneously. Specifically, it was thought that administering an HSP90 inhibitor with an agent known to block the function of a driver oncoprotein (a HSP90 client), could be especially effective [5].

Importantly, and in line with these predictions, Whitesell et al. have shown that co-administration of an HSP90 inhibitor substantially impairs the emergence of resistance to anti-estrogens in a model of estrogen receptor-positive human breast cancer [6]. Similarly, Smyth et al. have demonstrated that administering an HSP90 inhibitor with the BRAF inhibitor vemurafenib can overcome or delay the appearance of resistance to vemurafenib in models of mutant BRAF human melanoma [7] (Figure 1B).

Consistent with the established role of HSP90 in protein and morphological evolution, and in anti-fungal resistance [5], these new findings support calls for clinical testing of frontline combinations of HSP90 inhibitors with various molecularly targeted agents – to block the evolution of resistance and prevent the survival of the nastiest cells in human cancers.
